# Mechanical Behavior and Microstructure Evaluation of Quicklime-Activated Cement Kiln Dust-Slag Binder Pastes

**DOI:** 10.3390/ma17061253

**Published:** 2024-03-08

**Authors:** Minhui Hu, Tianwen Dong, Zhenglong Cui, Zhuo Li

**Affiliations:** School of Civil Engineering, Liaoning Technical University, Fuxin 123000, China; 18342836618@163.com (M.H.); cui0815@126.com (Z.C.); 17660108571@163.com (Z.L.)

**Keywords:** cement kiln dust, quicklime, mechanical behavior, microstructure, sustainable material

## Abstract

Cement kiln dust (CKD) is a by-product of cement production, which has the shortcomings of low utilization and high-temperature activation. This study combined CKD and slag as precursors for preparing pastes through quicklime activation under ambient conditions. The effects of quicklime and CKD content on the workability (flowability and setting time), macro-mechanical properties, and micro-structure of the CKD-slag binders were analyzed. The experimental results showed that the rapid precipitation of Ca^2+^, Si^4+^, and Al^3+^ ions from the CKD provided more nucleation sites for the formation of calcium aluminosilicate hydrate (C-(A)-S-H) gel and enhanced the reactivity of the binder system under the influence of the activator (CaO). The specimens had the highest unconfined compressive strength (UCS) (24.6 MPa) after 28 days with 10% quicklime content and 60% CKD content; scanning electron microscopy with energy-dispersive X-ray (SEM-EDX) analysis showed that the Ca/Si ratio of the C-(A)-S-H gel was minimized, leading to a denser microstructure and better binding ability under this mixing proportion. Therefore, this study may provide novel binder materials with a high proportion of CKD under ambient conditions.

## 1. Introduction

Cement kiln dust (CKD), a by-product generated from the cement manufacturing process, has an output ratio of around 5–20% of cement production [1,2]. In 2022, China’s cement production reached 21.18 billion tons, and the output of CKD exceeded 1.06 million tons. The primary method of collecting CKD is through electrostatic precipitators. There are three main methods for CKD treatment. First, it can be blended into the cement production process, which reduces the demand for raw materials. However, it is important to note that this method consumes a significant amount of energy [3]. Second, CKD can be used as an activator for alkali activation materials; however, the dosage required is small [4]. Finally, the majority of CKD is landfilled. The disposal of CKD increases the cost burden of the factory, occupies land resources, and contaminates ground water [5,6]. At present, the comprehensive utilization rate of CKD remains low.

CKD has a chemical composition similar to that of cement and is composed of fine alkaline particles [7]. It can be used as an activator or precursor to prepare cementitious materials. The chemical composition (alkali oxides, chlorides, and sulfates) of CKD will change between plants, leading to uncertainty in predicting the performance of CKD in cement products [8]. Additionally, the high chlorine and sulfate content will limit the recycling of CKD in building structures [9,10]. Therefore, the maximum amount of CKD used to replace cement in building structures should not exceed 10% [11,12]. However, in non-structural materials, CKD can be effectively utilized as a major component of cementitious material. In this context, the presence of free lime and sulfates may play an important role in the hydration reaction process [13,14]. Additionally, most studies have reported that CKD-based binders require heat curing to reach satisfactory strength. Ahmari et al. [15] investigated the durability properties of CKD-cemented copper mine tailings after curing at 90 °C for seven days. Their study revealed the formation of calcium-based products within the binder system. Yaseri et al. [16] studied geopolymers with a high-volume of CKD cured at a temperature of 60 °C for 24 h. They found that the 28-day compressive strength of the geopolymer paste, prepared with 45% CKD and 55% silica fume reached 27 MPa.

Alkali-activated materials are formed through the reaction of alkaline activators with industrial byproducts [17]. During the alkali activation process, the type of activator and the concentration of cations (Ca^2+^, Si^4+^, and Al^3+^) in the system will affect both the extent of the hydration reaction and the mechanical properties of the specimens. Activators can generally be divided into strong and weak alkaline activators. Strong alkaline solutions (e.g., NaOH, Na_2_SiO_3_) conducive to Si-O and Al-O bonds fracture in pozzolanic materials, thereby improving the reaction degree of binders. However, higher concentrations of alkaline solutions can reduce the flowability of the mixture [18,19]. Consequently, this approach will be prone to issues such as high viscosity, rapid setting, and late strength decline [20,21]. In contrast, quicklime is a weakly alkaline activator. Its hydration product, calcium hydroxide, creates an alkaline environment conducive to chemical reactions. Without the addition of a water-reducing agent, the mixture can obtain greater flowability and better mechanical properties. Furthermore, cations play a crucial role in the performance of alkali-activated materials [22]. Ca^2+^ precipitates into C-(A)-S-H gel during hydration, enhancing setting speed and early strength, while Si^4+^ and Al^3+^ can improve the reaction degree of the system.

Slag, an industrial waste material, has high reactivity at room temperature due to its elevated Ca^2+^ content [23]. Nevertheless, the slow strength development of slag paste activated by quicklime resulted from the initial pH being low. The addition of CKD increases alkalinity and leads to improved mechanical strength [24,25,26,27,28]. Furthermore, Lachemi et al. [29] found that the mixture, with a CKD/slag ratio of 6, met the requirements for controlled low-strength materials (CLSM) and demonstrated better performance than CKD-modified CLSM mixtures. A study by Chaunsali et al. [24] found that the addition of CKD to a cementing material became the main reason for strength gain due to the formation of gels. CKD-slag pastes achieved significant strength gains in hot curing and wet curing conditions. The strength of the specimen after 28 days of curing in saturated lime water was higher than after 48 h of hot curing at 75 °C. However, there are few studies that use quicklime as an activator to prepare CKD-slag binders at room temperature. Moreover, the hydration in calcium-rich systems is more complex than the hydration of cement, and further clarification of the reaction mechanism for binder materials with a high dosage of CKD is needed.

Based on previous studies, a combination of CKD and slag was selected as the precursor, with quicklime used as an activator to prepare binder pastes. Comparative analysis of the differences in hydration products at different CKD contents was carried out using microstructure testing methods such as X-ray diffraction (XRD), thermogravimetric analysis (TGA), Fourier transform infrared (FTIR) spectroscopy, and scanning electron microscopy with energy dispersive X-ray (SEM-EDX) analysis. The effects of quicklime content (5–20%) and CKD content (20–80%) on the flowability, setting time, and mechanical properties of CKD-slag binder pastes were systematically investigated. In this study, the production of high CKD volume binder materials under standard curing conditions was realized, providing a novel method for treating solid waste and preparing low-cost binders.

## 2. Materials and Methods

### 2.1. Materials

**Figure 1:**
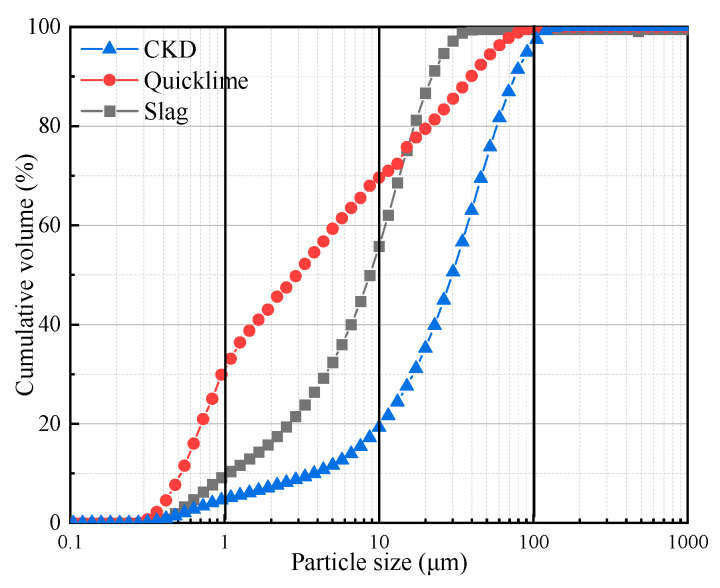
Particle size distribution of quicklime, slag, and CKD.

**Figure 2:**
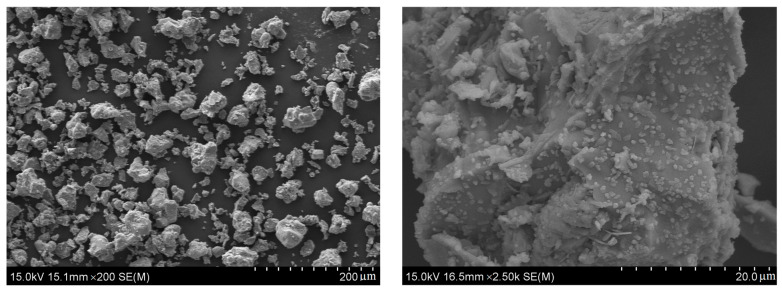
SEM images of the CKD powder.

The materials used in this study were CKD, slag, and quicklime. CKD was obtained from the Daying cement factory in Fuxin, Liaoning Province, China; the S95 grade slag was produced by the Borun Casting Material Limited Company located in Gongyi, Henan Province, China; and the quicklime was obtained from a local lime factory in Fuxin, Liaoning Province, China. Figure 1 shows the particle size distributions of the raw materials tested by a laser diffraction particle size analyzer (Mastersizer 2000, Malvern Panalytical Ltd., Malvern, UK). According to the analysis results listed in Table 1, the D_v_(90) of CKD, slag, and quicklime were 40.98, 11.68, and 13.08 μm, respectively. This indicates that CKD had the coarsest particle size among all three materials. In addition, the CKD particles had the largest difference between the values of D(3,2) and D(4,3), suggesting that the shape of CKD particles was irregular (see Figure 2). Furthermore, the compositions and proportions of materials were tested by an X-ray fluorescence (XRF) spectrometer (XRF-1800, Shimadzu, Japan), with the results shown in Table 2.

### 2.2. Preparation of the Specimens

The binder phase consisted of CKD, slag, quicklime, and water. Four different weight ratios (5%, 10%, 15%, and 20%) of quicklime to the total mixture and seven different proportions of CKD (20%, 30%, 40%, 50%, 60%, 70%, and 80%) were used in this study. The mixing proportion design scheme is shown in Table 3. First, CKD, slag, and quicklime were placed in a blender and mixed slowly for 3 min. Subsequently, water was added to the mixtures, and the mixing was continued for an additional 5 min. The resulting uniform slurry was poured into plastic molds measuring 40 mm × 40 mm × 40 mm and vibrated for 2 min to eliminate air. Finally, the specimens were placed in a standard curing chamber and demolded after 24 h.

### 2.3. Experimental Methods

#### 2.3.1. Flowability and Setting Time

According to GB/T8077-2012 [30], the flow degree of paste was tested with a copper cone. The flow value was determined as the average diameter in the vertical direction of the paste. Furthermore, GB/T 1346-2011 [31] was used to determine the setting time. The penetration time of the Vicat Needle sank into the paste 0.5–1.0 mm away from the bottom plate, which was determined as its initial setting time. The time at which the needle sank into the paste no more than 1.0 mm was the final setting time.

#### 2.3.2. Unconfined Compression Test

According to ASTM D4219-08 [32], the change in unconfined compressive strength (UCS) of the hardened paste was analyzed after curing for 7, 14, and 28 days. A WDW300E (Jinan Times testing machine Co., LTD, Shandong, China) testing machine with displacement loading was used to conduct the UCS test. It was loaded at 0.5 mm/min. Then, 5-mm steel plates were placed on the upper and lower surfaces of the specimen to ensure that the force on the machine was uniform.

#### 2.3.3. Hydration Product Tests

All specimens were broken with a steel hammer after the 28-day UCS test and experienced termination of the hydration reaction. The fragments were ground into powders for XRD, TGA, FTIR, and SEM-EDS tests. Each specimen was individually sealed to avoid wetting and carbonation.

1. The mineral composition of the CKD and binder pastes were analyzed using XRD (XRD instrument model: Rigaku Ultima IV and 1.5418 Å wavelength). The parameters set during the tests had a 0.02 °C step size with a test interval ranging from 10° to 80° and a scan speed of 2°/min.
2. The mass changes of reaction products at various temperatures were measured using the TGA (TGA instrument model: TGA/DSC-1, Mettler Toledo, Greifensee, Switzerland) method. The heating temperatures were increased from 30 °C to 1000 °C at a rate of 10 °C/min, and the heating atmosphere was nitrogen. The DTG results were derived from the first derivative of the weight loss data.
3. FTIR was used to obtain information regarding the molecular structure and chemical bonds of the materials, and the wavenumber range for sample collection was 400 cm^−1^ to 4000 cm^−1^ (FTIR instrument model: Nicolet 10, Thermo Fisher Scientific, Dreieich, Germany).
4. The morphology of the hydration product was captured by SEM (SU-70, Hitachi High-tech Corporation, Tokyo, Japan) after 5000 times magnification, and their basic composition was studied by EDS (AZtec, Oxford instruments, Abingdon, UK).

## 3. Results

### 3.1. Flowability and Setting Time

**Figure 3:**
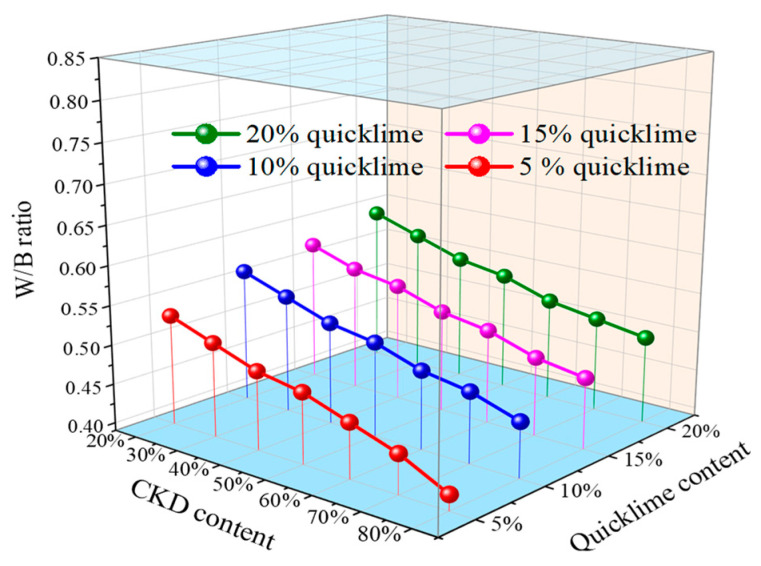
W/B ratio of the CKD-slag binder pastes.

The water/binder (W/B) ratio results of the CKD-slag binder pastes with different contents of quicklime and CKD were analyzed, as shown in Figure 3. To achieve the targeted flow (200 ± 10 mm), the required amount of water increased with higher quicklime dosage. When the CKD content was 80%, the W/B ratio of the binder paste with 5% quicklime was 0.41. As the quicklime content increased to 20%, the W/B ratio reached 0.5. The consumption of free water was generally the main factor increasing these W/B ratios because the calcium oxide in quicklime reacted with water and formed calcium hydroxide, consuming large amounts of free water. More quicklime increased the amount of calcium hydroxide, resulting in a higher W/B ratio. In addition, when increasing the CKD dosage, the W/B ratio of the fresh paste decreased. This was mainly due to the larger size of CKD particles compared to those of slag and quicklime (Figure 1), which required less water. Additionally, the higher CKD content meant less slag content, leading to a smaller W/B ratio. Other studies observed that increased slag dosage in the binder required additional water content to obtain the target flow value. For example, Liu et al. [33] found that the standard consistency water demand increased from 28.6% for pure OPC to 35.5% with a 50% slag mixture.

**Figure 4:**
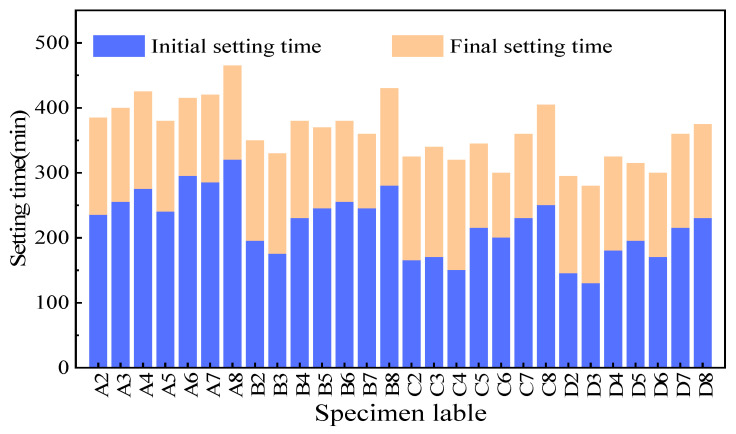
Setting time of the CKD-slag binder pastes.

The setting time of the fresh pastes with different quicklime and CKD dosages is shown in Figure 4. According to this figure’s findings, the setting time decreased with increased quicklime and CKD addition. When the quicklime content was 5% and the CKD content was increased from 20% to 80%, the initial setting time of the CKD-slag binder pastes was reduced by 31%, and the final setting time was reduced by 39%. When the CKD content was 80% and the quicklime content was increased from 5% to 20%, the initial setting time of the binder pastes was reduced by 28%, and the final setting time was reduced by 19%. In this case, soluble Ca from the quicklime precipitated as calcium hydroxide under alkaline conditions, which improved the hydration rate and formed calcium aluminosilicate hydrate (C-(A)-S-H) gel, thus shortening the setting time [34]. Moreover, Figure 3 shows that the W/B ratio decreased with increasing CKD dosage, suggesting that water reduction in the binder system was another factor that decreased the setting time.

### 3.2. Unconfined Compressive Strength

**Figure 5:**
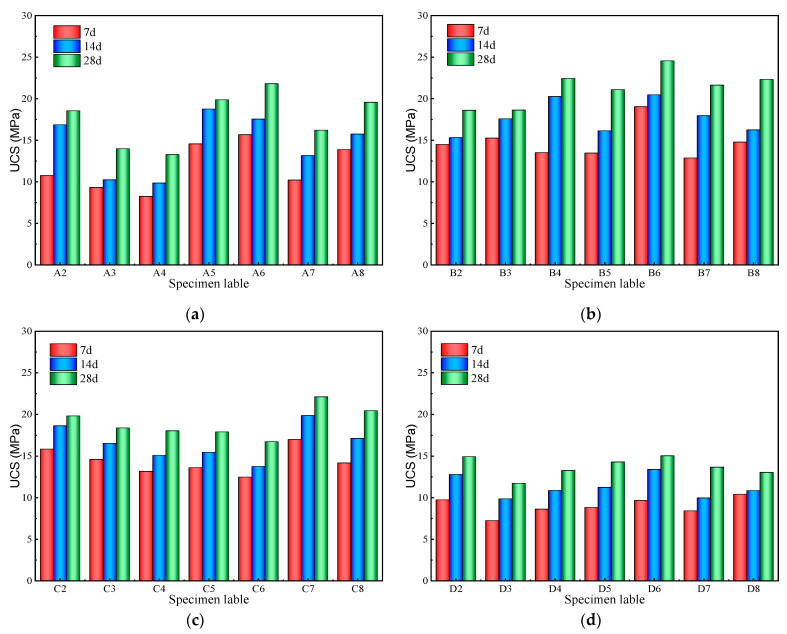
UCS of the CKD-slag binder pastes: (**a**) 5% quicklime; (**b**) 10% quicklime; (**c**) 15% quicklime; (**d**) 20% quicklime.

Figure 5a–d presents the unconfined compressive strengths (UCS) of the hardened pastes under different quicklime contents. With increased curing age, the UCS of the CKD-slag binder pastes increased gradually. However, the UCS of the specimen increased when the quicklime content was 10%, and the UCS decreased when the quicklime content exceeded 10%. The UCS for the specimens prepared with 10% quicklime content were 12.8%, 47%, and 64% higher than those made with 5%, 15%, and 20% quicklime content, respectively. The specimens reached the maximum UCS corresponding to 60% CKD content. These results are similar to the experimental results of Chaunsali et al. [24] regarding the optimal CKD content of 70% for CKD-slag binders.

**Figure 6:**
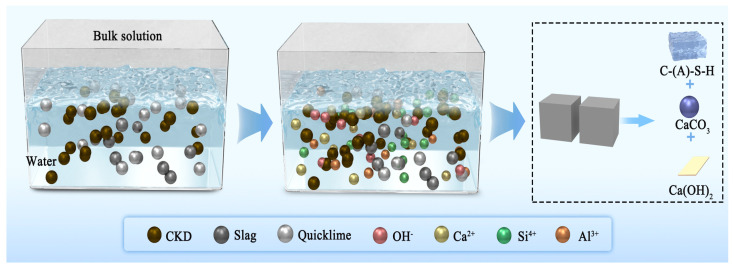
Schematic diagram of the CKD-slag binder pastes.

The UCS of the CKD-slag binder pastes mainly depended on three factors. First, quicklime, as an activator, produced calcium hydroxide by rapid hydration in contact with water, and the dissolved OH^−^ promoted the release of Ca^2+^, Si^4+^, and Al^3+^ in CKD, as shown in Figure 6. Second, as the main source of aluminosilicates, the silica and alumina in CKD formed C-(A)-S-H gels after hydration. Third, the calcium carbonate in the hydration products exhibited a filler effect and reduced porosity. The CKD-slag binder pastes showed a denser microstructure. When the amount of quicklime and CKD was too small, the amount of OH^−^ in the binder system was limited, the hydration degree of CKD was insufficient, and the hydration products were small. As the quicklime and CKD content increased, a sufficient amount of OH^−^ promoted the dissolution of cations in CKD. The hydration was more rapid and the amount of C-(A)-S-H gel increased, which is conducive to the improvement of UCS. However, when the amount of quicklime and CKD content was excessive, the gel generated in the early reaction wrapped around the surface of the CKD particles, preventing hydration. Meanwhile, excessive calcium hydroxide led to higher porosity, resulting in the corresponding UCS decreasing significantly. The hydration mechanism of the CKD-slag binder pastes will be further analyzed in the following sections.

### 3.3. XRD Analysis

**Figure 7:**
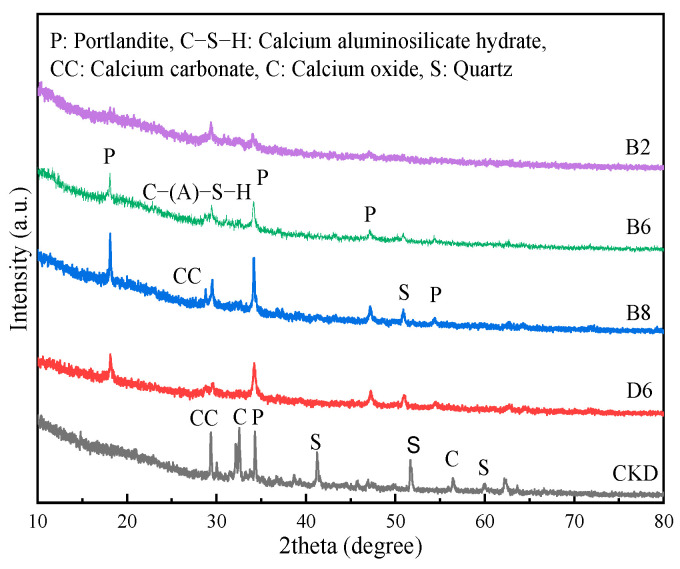
XRD patterns of the CKD and paste specimens.

Figure 7 shows the XRD patterns of the CKD powder and specific specimens after 28 days. According to Figure 7, CKD was mainly composed of calcite (CaCO_3_), lime (CaO), quartz (SiO_2_), and portlandite (Ca(OH)_2_). The peak at 25–40° was the main reactive phase in hydration, forming amorphous gels [35]. New portlandite was detected at 18.1°, 47.1°, and 54.3°, which was attributed to the hydrolysis reaction of CKD and quicklime. Notably, the diffraction peaks of portlandite clearly strengthened with increasing CKD content from 20% to 80%, and the quartz peaks in the CKD powder were detected at 41.2°, 51.7°, and 60° in the XRD patterns. After hydration, the diffraction peaks at 41.2° and 60° disappeared, and the diffraction peak at 51.7° diminished, indicating the breaking of the Si–O bonds in the alkaline environment.

In contrast to the results of Chaunsali et al. [24], no evidence of ettringite was found in Figure 7 because of the low sulphate content in CKD (Table 2). The lime peaks at 32.3°and 56.5° in the CKD powder completely disappeared, suggesting that Ca^2+^ was integrated into the binder structure. In addition, free Ca^2+^ acted as a charge balancer for alumina, forming C-(A)-S-H gel during hydration [36]. However, when the quicklime content was 20%, the diffraction peaks corresponding to portlandite and calcite in the D6 specimen experienced a reduction. Because excessive portlandite increased porosity, the portlandite and calcite became physically encapsulated within the pores. Moreover, the XRD patterns of the CKD-slag binder pastes showed no noticeable difference, indicating that the long-range order of the pastes did not change regardless of the CKD content.

### 3.4. TG Analysis

**Figure 8:**
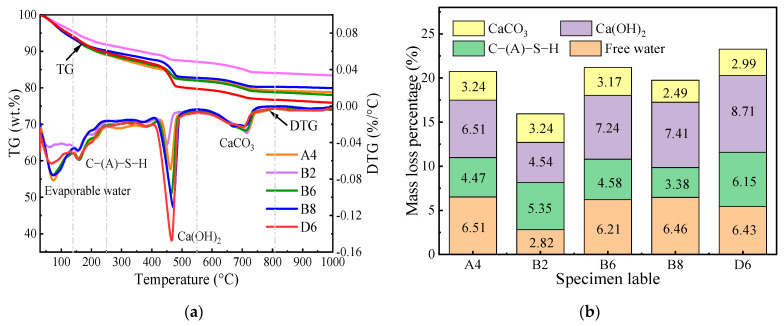
(**a**) TG-DTG patterns and (**b**) rate of mass change of the CKD-slag binder pastes.

Figure 8a presents the TG-DTG curves of CKD-slag binder pastes. The specimens with different quicklime and CKD dosages showed significant mass changes at 400–500 °C. Increasing the quicklime proportion from 5% to 20% led to an increase in mass loss from 21.1% to 24.1%. Previous research [37,38] has found that the mass change in the DTG curves between 30 °C and 200 °C is the dehydration of the gel. In this case, the peak between 30–130 °C was the loss of evaporable water, while the mass loss peak of interlayer water was observed between 130 and 200 °C. In addition, the peak between 370 and 470 °C indicated the de-hydroxylation of Ca(OH)_2_, and the significant peak above 600 °C came from the decomposition of calcite. Decomposition peaks of portlandite became more distinct with increased quicklime content. As discussed above, excess Ca(OH)_2_ increased the porosity, and some hydration products were encapsulated within the pores, confirming the above XRD results.

Figure 8b shows the mass loss percentages of hydration products calculated for different peak temperature ranges. Notably, the weight loss percentage of C-(A)-S-H and Ca(OH)_2_ was lower than in the other pastes with 20% CKD content, indicating that insufficient hydration products formed, leading to a smaller UCS. The binder pastes with 10% added quicklime contained 4%, 5.58%, and 3.38% C-(A)-S-H corresponding to CKD dosages of 20%, 60%, and 80%, respectively. With increasing CKD content, more soluble silica and alumina were incorporated into the system. Thus, a greater amount of C-(A)-S-H gel was formed, making the microstructure denser, which effectively improved the UCS of the binder pastes [39]. However, when the amount of CKD was too large, the C-(A)-S-H gels that formed at early hydration wrapped the CKD particles due to the rapid reaction rate, which stopped the hydration of the source material. In addition, the amount of gels increased with quicklime content, mainly because quicklime enhanced the degree of reaction within the binder system, generating larger quantities of hydration products. The Ca(OH)_2_ mass loss percentages of the A4, B6, and D6 specimens were 6.51%, 7.24%, and 8.71%, respectively. However, when more than the optimum amount of Ca(OH)_2_ was generated, the porosity of the binder pastes increased, causing a reduction in UCS. Moreover, Sharma et al. also observed that carbonation curing resulted in more calcite production in cement-CKD mortar, with reduced porosity and increased strength. Figure 8b shows that the weight loss of calcite in the B8 and D6 specimens was lower than the other pastes, suggesting that an insufficient amount of calcite for pore filling was another reason for the decline in UCS. Therefore, the binder paste prepared with 10% quicklime and 60% CKD produced higher amounts of C-(A)-S-H gel and calcite during hydration, which reduced porosity and increased compressive strength.

### 3.5. FTIR Analysis

**Figure 9:**
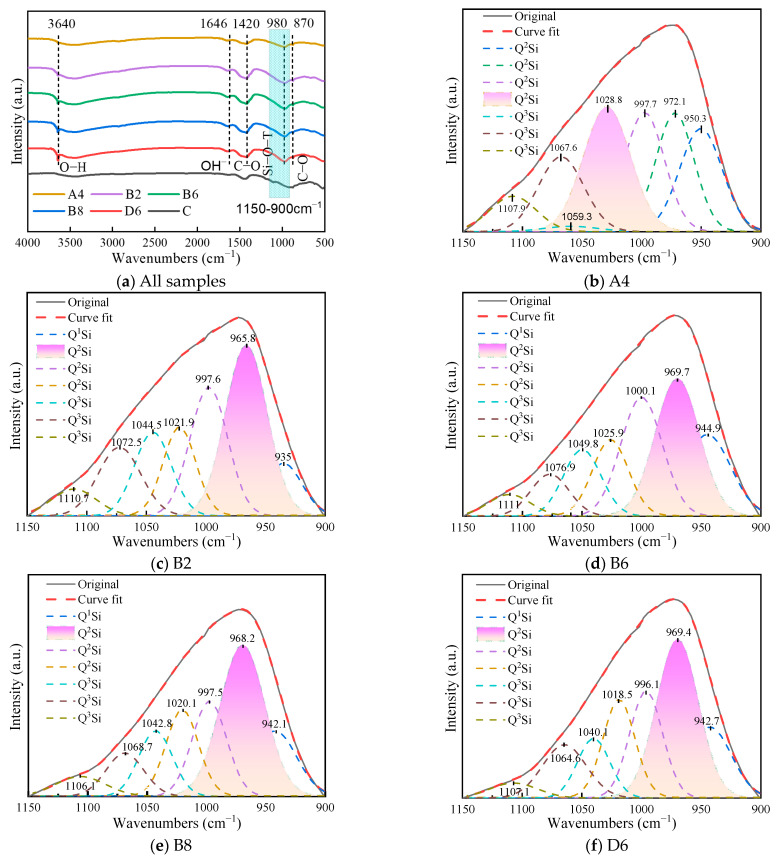
FTIR curves and main peaks curve fitting of the CKD-slag binder pastes.

Figure 9a–f displays the FTIR analyses of CKD powder and the binder pastes after 28 days. The absorption bands of CKD powders around 980 and 1420 cm^−1^ were dominated by T-O-Si (T = Al, Si) stretching vibrations and O-C-O vibrations in carbonates [40,41]. The band around 980 cm^−1^ in the CKD powder shifted to the left after hydration, transiting into stronger atomic bonds. This indicated that C-(A)-S-H gels were formed following the dissolution of aluminosilicates. Additionally, paste specimens showed narrower T-O bands than CKD powder. This is mainly because the amorphous silica and alumina in raw material formed a more orderly structure after dissolution. In the binder paste specimens, the band around 1646 cm^−1^ was the vibration of the H-O-H about bound water in the gel [42]. Furthermore, the band near 3640 cm^−1^ was the O-H stretching vibration in Ca(OH)_2_ [43]. The intensity of O-H in this region strengthened with higher quicklime and CKD contents, indicating that more Ca(OH)_2_ formed, which agreed with the TG-DTG results.

For further analysis of the differences in gels between different specimens, a Gaussian function was used to fit the peaks at 900–1150 cm^−1^. The absorption peaks were divided into seven distinct peaks, as shown in Figure 9b–f. The fitted wavenumbers of the main peaks ranged from 969 to 1028 cm^−1^, which corresponded to the absorption peak of the C-(A)-S-H gel [44]. According to the literature, the bands at 840 cm^−1^–1300 cm^−1^ in Table 4 are divided into five forms of [SiO_4_] [45,46]. For the CKD-slag binder pastes, the main existing form of [SiO_4_] of the C-(A)-S-H gel was entirely Q^2^Si, suggesting that the hydration degree of the product remained unchanged with increased CKD content. The wavenumber of the main peaks increased from 965.8 to 969.7 cm^−1^ and then decreased to 968.2 cm^−1^ with increasing CKD content. This demonstrated that the reactivity of the binder system was maximum at 60% CKD content. By comparing specimens A4, B6, and D6, the relative area of the absorption peaks increased from 23.63% to 31.26%, indicating that the reactivity of the binder system effectively increased with increasing quicklime content.

### 3.6. SEM-EDS

**Figure 10:**
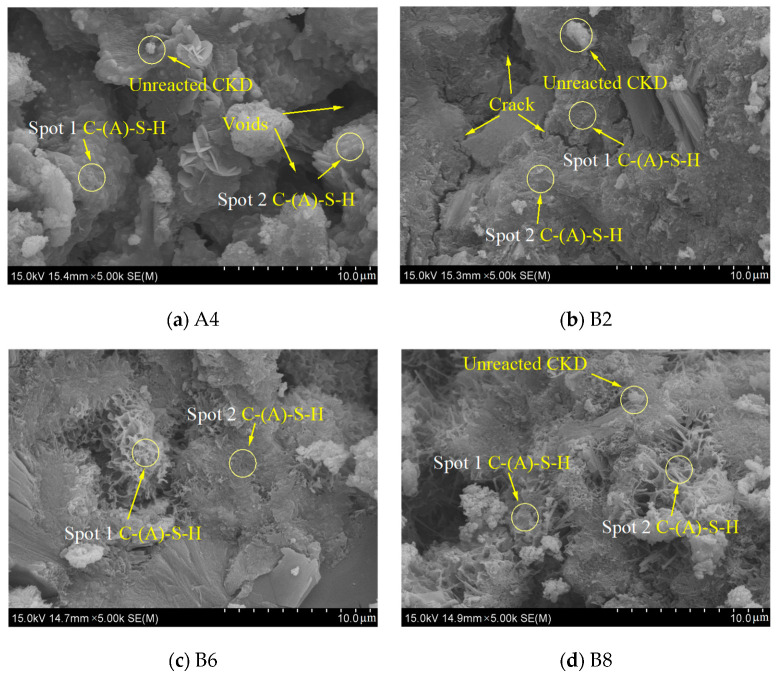

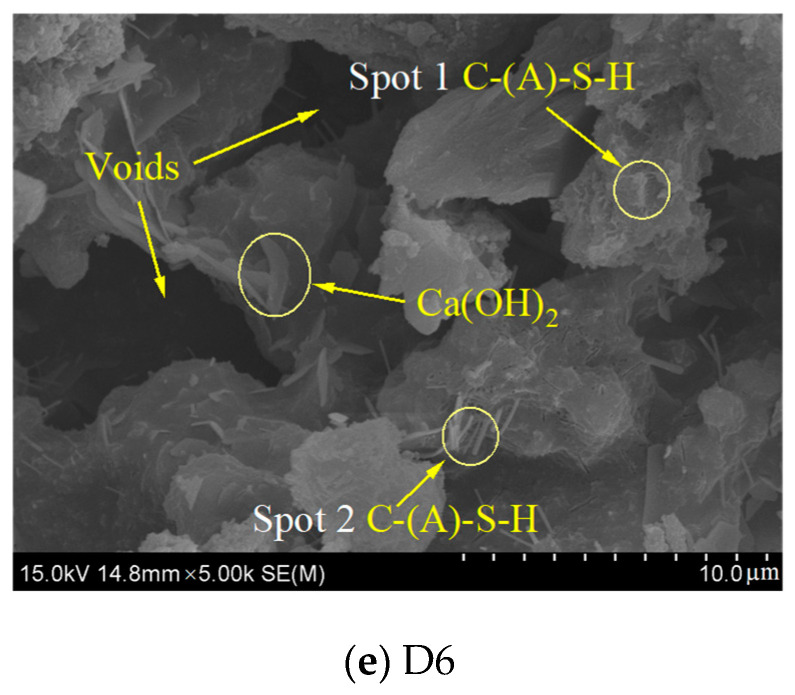
SEM images of the CKD-slag binder pastes after 28 days.

The micromorphology of the binder pastes after 28 days is shown in Figure 10a–e. The binder particles of A4 were presented with a significant amount of pores, as shown in Figure 10a. The lower 5% hydrated quicklime provided a limited amount of OH^−^ in the binder system and an insufficient amount of hydration products. When the quicklime content was 10%, the CKD particles were held together by gel, forming a dense microstructure in the specimens with 20%, 60%, and 80% CKD content, as shown by Figure 10b–d. For a given quicklime dosage of 20%, significantly less gel and more Ca(OH)_2_ were observed (Figure 10e). Ca(OH)_2_ released a significant amount of heat, producing pores during hydration [47]. Although CaCO_3_ was present in the XRD and TG analyses, it was almost undetectable in the SEM images. This was mainly because the CaCO_3_ particles filled the pores, resulting in a denser porous structure.

Table 5 presents the chemical compositions of the two spots (spots 1 and 2) in the above SEM images, as determined from the EDX spectra. Overall, the observed hydration product is C-S-H gel and integrated aluminum. With increased CKD content, the Si/Al ratio of the specimens increased, leading to a more rigid C-(A)-S-H gel. This confirms the enhancing effect of CKD on solubility. The Ca/Si ratio in the gel is usually considered to be in the range of 1.5 to 2 [48], while the EDS results for specimens with 5% and 20% quicklime content greatly exceeded this range, indicating that the unreacted calcium-based compounds in the system were encapsulated within the hydration product. When the quicklime content was 10%, the Ca/Si ratio of the specimens with 20%, 60%, and 80% CKD content decreased from 1.7 to 1.5 and then increased to 2.3, suggesting that the difference among the three specimens was in the compactness of the formed C-(A)-S-H gel. With 60% CKD content, the Ca/Si ratio was the smallest, indicating that the specimen had the best reactivity [49]. Calcium, the primary component in the paste matrix, was fully utilized to form C-(A)-S-H gel, which was similar to the FTIR peak fitting results. Furthermore, the experimental results of Abdel-Gawwad et al. [4] and Manzano et al. [50] showed that specimens with a lower Ca/Si ratio had higher binding capacity and better mechanical properties than those with a higher Ca/Si ratio.

## 4. Conclusions

This study explored the feasibility of CKD-based binder materials. The binder pastes were prepared using a combination of CKD and slag as a precursor, alongside quicklime activation under ambient conditions. The fluidity, setting time, and mechanical behavior of the binder pastes with different quicklime and CKD contents were then investigated. Additionally, the microscopic performance of the CKD-slag binder materials was analyzed using XRD, TG, FTIR, and SEM-EDS techniques. The following are the main conclusions:

(1) The UCS enhancement of CKD-slag binders can be attributed to three main factors: (a) The rapid dissolution of quicklime, which produced calcium hydroxide, and the dissolved Ca^2+^ improved the alkaline environment, which promoted the precipitation of Ca^2+^, Si^4+^, and Al^3+^ ions from CKD. (b) Silica and alumina in CKD served as primary sources of aluminosilicates, forming C-(A)-S-H gel after hydration. (c) Calcium carbonate created a micro-aggregate effect, filling the pores and reducing the porosity.

(2) The setting time was reduced by adding more CKD content, accelerating the hydration. Conversely, higher quicklime content led to a larger W/B ratio due to the rapid dissolution of quicklime, consuming a large amount of free water and increasing the system’s water requirements.

(3) TG and FTIR analysis revealed that the CKD content did not change the long-range order of the hydration products. The predominant form of [SiO_4_] in C-(A)-S-H gel was Q^2^Si. The reactivity initially increased with an increase in CKD content, reached a peak, and subsequently decreased. SEM-EDS analysis of the C-(A)-S-H composition revealed that the Ca/Si ratio was the lowest at a 60% CKD content, indicating a high binding capacity and a significant enhancement in the UCS of the specimens.

Therefore, this paper successfully developed novel CKD-based binder materials. However, the reactivity of the CKD-slag binders is observed to be relatively low, and the C-(A)-S-H gel production is insufficient. Future research should focus on enhancing the reactivity of the precursor by analyzing its chemical composition and adding admixtures. A more comprehensive study of CKD-slag binders is essential to provide a reliable method for the consumption and treatment of solid waste.

## Figures and Tables

**Table 1:** Particle size analysis of CKD, slag, and quicklime.

Element	CKD	Slag	Quicklime
D_v_(10) ^1^	4.37	1.14	0.60
D_v_(50)	34.20	9.89	1.98
D_v_(90)	87.11	24.89	45.40
D(3,2) ^2^	7.34	3.46	1.48
D(4,3) ^3^	40.98	11.68	13.08

^1^ D_v_(10), D_v_(50), and D_v_(90) represent the particle size values larger than the cumulative particle size distributions of 10%, 50%, and 90%. ^2^ Surface volume mean diameter. ^3^ Volume mean diameter.

**Table 2:** Composition and content of CKD, slag, and quicklime (wt.%).

Chemical Composition	CaO	SiO_2_	Al_2_O_3_	MgO	Fe_2_O_3_	K_2_O	Na_2_O	SO_3_	LOI
CKD	83.20	6.21	0.61	0.13	6.36	1.20	0.14	0.95	23.65
Slag	68.22	12.61	5.95	3.85	2.05	0.62	0.21	0.80	1.32
Quicklime	98.21	0.18	0.10	1.03	0.40	-	-	0.08	8.37

**Table 3:** Mixing proportions of the CKD-slag binder pastes.

ID	Quicklime (%)	CKD (%)	Slag (%)	W/B Ratio	Flow (mm)	Initial Setting Time (min)	Final Setting Time (min)
A2	5	19	76	0.53	203	235	385
A3	5	29	66	0.51	200	255	400
A4	5	38	57	0.49	198	275	425
A5	5	48	47	0.48	201	240	380
A6	5	57	38	0.46	206	295	415
A7	5	67	28	0.44	200	285	420
A8	5	76	19	0.41	197	320	465
B2	10	18	72	0.56	201	195	350
B3	10	27	63	0.54	203	175	330
B4	10	36	54	0.52	198	230	380
B5	10	45	45	0.51	200	245	370
B6	10	54	36	0.49	202	255	380
B7	10	63	27	0.48	199	245	360
B8	10	72	18	0.46	203	280	430
C2	15	17	68	0.57	204	165	325
C3	15	26	59	0.55	202	170	340
C4	15	34	51	0.54	200	150	320
C5	15	43	42	0.52	198	215	345
C6	15	51	34	0.51	201	200	300
C7	15	60	25	0.49	203	230	360
C8	15	68	17	0.48	200	250	405
D2	20	16	64	0.59	200	145	295
D3	20	24	56	0.57	202	130	280
D4	20	32	48	0.55	199	180	325
D5	20	40	40	0.54	201	195	315
D6	20	48	32	0.52	203	170	300
D7	20	56	24	0.51	201	215	360
D8	20	64	16	0.50	202	230	375

**Table 4:** Five existing forms of [SiO_4_] according to Ref. [45].

Wavenumber (cm^−1^)	Forms	Characteristic
840–900	Q^0^Si	Four non-bridge oxygen in [SiO_4_]
900–950	Q^1^Si	Three non-bridge oxygen in [SiO_4_]
950–1030	Q^2^Si	Two non-bridge oxygen in [SiO_4_]
1030–1120	Q^3^Si	One non bridge oxygen in [SiO_4_]
1120–1300	Q^4^Si	Completely polymerized [SiO_4_]

**Table 5:** Atomic ratio of spots 1 and 2 in Figure 10.

ID	Region	Elements (Atomic (%))							
		Ca	Si	Al	Na	S	Ca/Si	Ca/Al	Si/Al
A4	Spot 1	35.00	4.40	1.76	0.00	0.00	7.95	19.89	2.50
	Spot 2	31.26	13.7	4.14	1.24	0.00	2.28	7.55	3.31
B2	Spot 1	17.23	10.15	4.59	1.69	1.76	1.70	3.75	2.21
	Spot 2	16.04	9.67	6.05	1.9	1.16	1.66	2.65	1.60
B6	Spot 1	19.84	14.33	3.98	0.94	1.01	1.38	4.98	3.60
	Spot 2	16.14	10.21	4.95	0.96	0.98	1.58	3.26	2.06
B8	Spot 1	32.65	13.11	0.65	0.00	0.88	2.49	50.23	20.17
	Spot 2	18.37	9.06	1.47	0.89	1.53	2.03	12.50	6.16
D6	Spot 1	34.74	2.15	0.00	0.00	0.74	16.16	0.00	0.00
	Spot 2	40.57	2.72	1.94	0.00	0.65	14.92	20.91	1.40

## Data Availability

Data will be made available upon request.
